# Supplementary material for: Immune Effects of the Nitrated Food Allergen Beta-Lactoglobulin in an Experimental Food Allergy Model
Source: Nutrients. 2019 Oct 15;11(10):2463. doi: 10.3390/nu11102463 (PMC6835712; doi:10.3390/nu11102463)
Supplement: Supplementary file 1 [file nutrients-11-02463-s001.zip › Supporting Information 1.pdf]

A

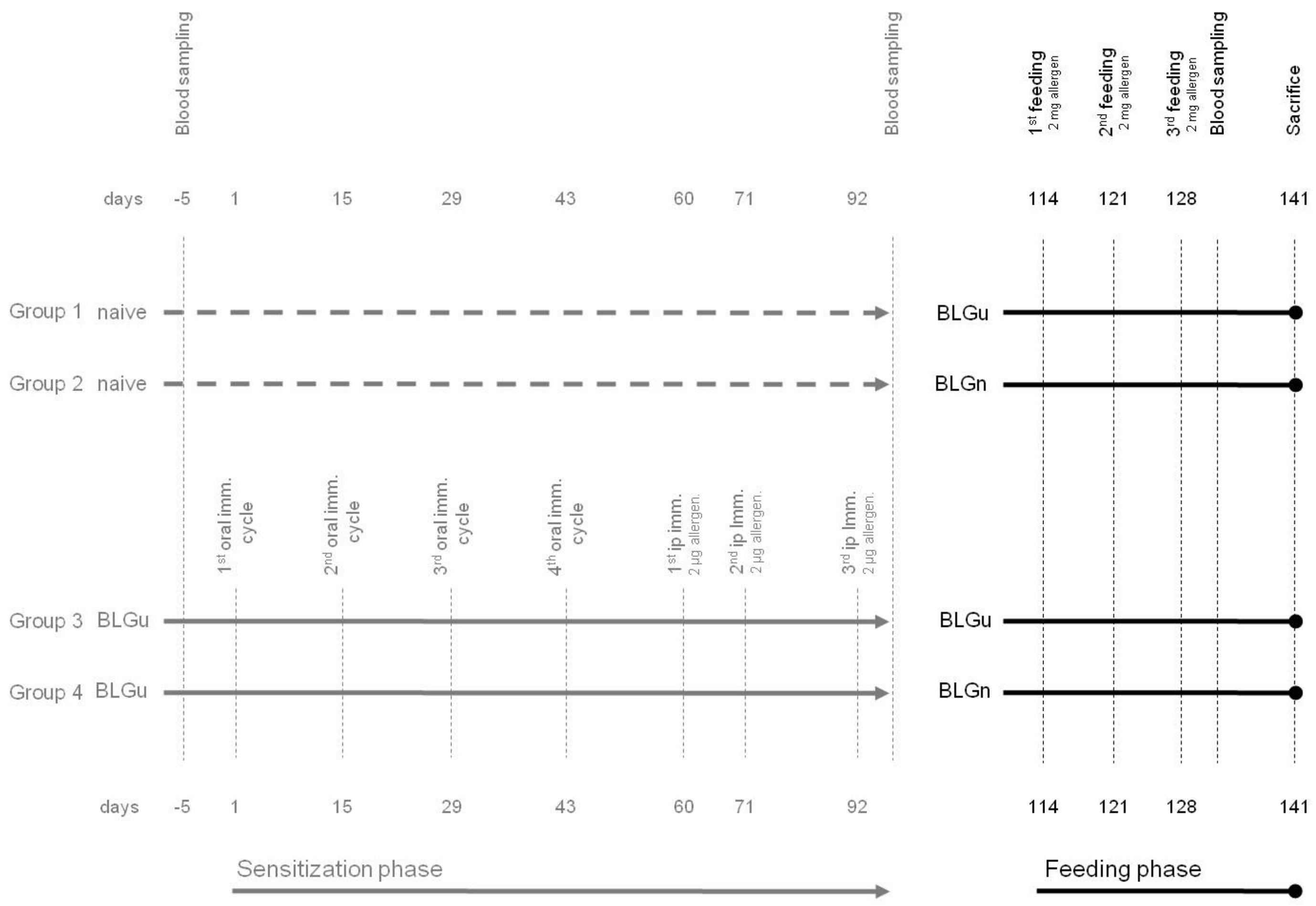

B

Oral immunization cycle

day	pre-treatment	allergen gavage
1	116 µg PPI iv	
2	116 µg PPI iv	200 µg BLGu + 2 mg sucralfate ig
3	116 µg PPI iv	200 µg BLGu + 2 mg sucralfate ig

Supporting Information 1

Schematic depiction of mouse experiments. (A) Feeding protocol of naïve mice (groups 1 and 2) and treatment protocol for allergy induction and subsequent feedings. **(B)** Detailed schedule and protocol of oral immunization cycles. BLG, beta-lactoglobulin; BLGn, nitrated BLG; BLGu, untreated BLG; PPI, proton pump inhibitor; ip, intraperitoneal
