# Supplementary material for: Immune Effects of the Nitrated Food Allergen Beta-Lactoglobulin in an Experimental Food Allergy Model
Source: Nutrients. 2019 Oct 15;11(10):2463. doi: 10.3390/nu11102463 (PMC6835712; doi:10.3390/nu11102463)
Supplement: Supplementary file 1 [file nutrients-11-02463-s001.zip › Supporting Information 2.pdf]

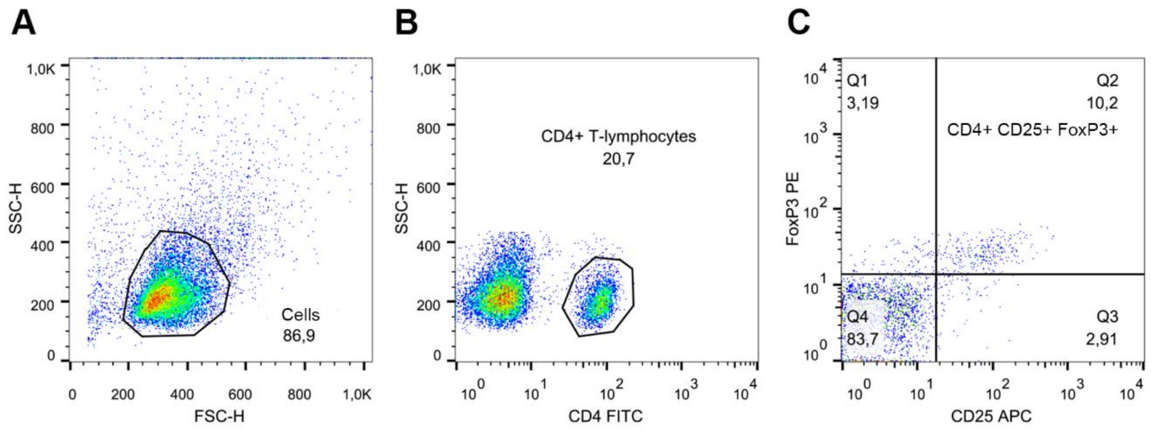

Supporting Information 2

Exemplary gating strategy of Tregs from isolated spleen cells. (A) Cells were identified according to forward (FSC) and side scatter (SSC) characteristics. **(B)** CD4+ lymphocytes were gated and **(C)** analyzed for CD25 and FoxP3. Cells positive for all three markers were identified as Tregs. Numbers indicate percentage of gated cells. FSC, forward scatter; SSC, side scatter; Tregs, regulatory T cells; Q, quadrant
