# Supplementary material for: Immune Effects of the Nitrated Food Allergen Beta-Lactoglobulin in an Experimental Food Allergy Model
Source: Nutrients. 2019 Oct 15;11(10):2463. doi: 10.3390/nu11102463 (PMC6835712; doi:10.3390/nu11102463)
Supplement: Supplementary file 1 [file nutrients-11-02463-s001.zip › Supporting Information 3.pdf]

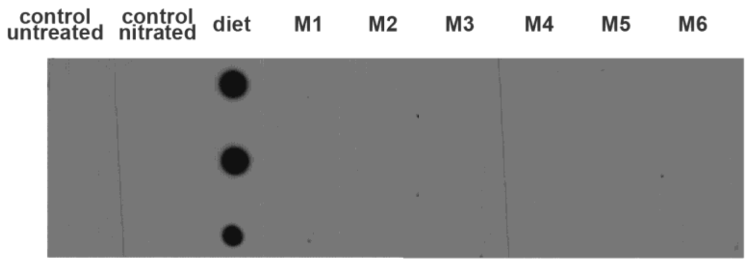

Supporting Information 3

Control membrane for unspecific antibody staining in dotblot experiments. Stomach content dotted on a second membrane was incubated only with HRP-linked goat anti rabbit IgG (1:5000 in dilution buffer) to control for unspecific binding of the secondary antibody. Mouse feed displayed major unspecific binding. M, mouse; HRP, horseradish peroxidase
