# Supplementary material for: Immune Effects of the Nitrated Food Allergen Beta-Lactoglobulin in an Experimental Food Allergy Model
Source: Nutrients. 2019 Oct 15;11(10):2463. doi: 10.3390/nu11102463 (PMC6835712; doi:10.3390/nu11102463)
Supplement: Supplementary file 1 [file nutrients-11-02463-s001.zip › Supporting Information 4.pdf]

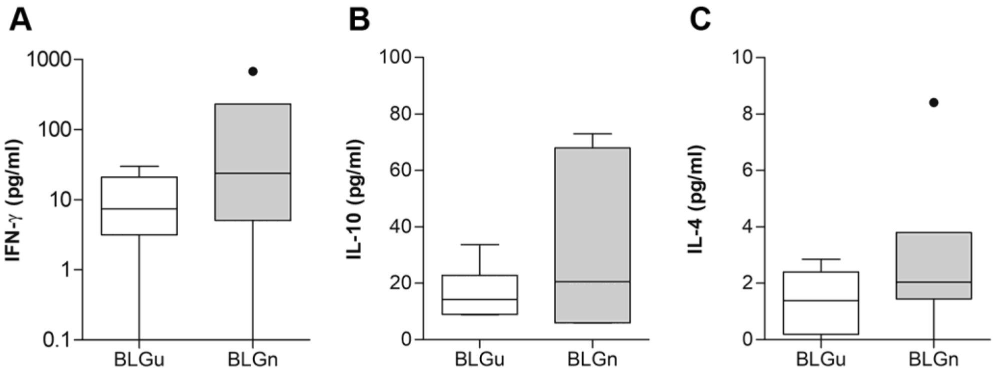

Supporting Information 4

Effect of re-stimulation of spleen cells with BLGn and influence of prior feeding on cytokine production in naïve mice. Isolated spleen cells from non-allergic mice def with either Blgu or BLGn were stimulated with BLGn to evaluate cytokine production upon allergen encounter. Baseline levels were subtracted. We could not detect significant differences between feeding groups for **(A)** IFN-γ (Mann-Whitney *U* test) or **(B)** IL-10 (unpaired t test) compared to group BLGu. **(C)** IL-4 remained mostly below detection limit of the assay used and did not show any differences (Mann-Whitney *U* test). * $p<0.05$; BLG, beta-lactoglobulin; BLGn, nitrated BLG; BLGu, untreated BLG
