# Supplementary material for: Immune Effects of the Nitrated Food Allergen Beta-Lactoglobulin in an Experimental Food Allergy Model
Source: Nutrients. 2019 Oct 15;11(10):2463. doi: 10.3390/nu11102463 (PMC6835712; doi:10.3390/nu11102463)
Supplement: Supplementary file 1 [file nutrients-11-02463-s001.zip › Supporting Information 5.pdf]

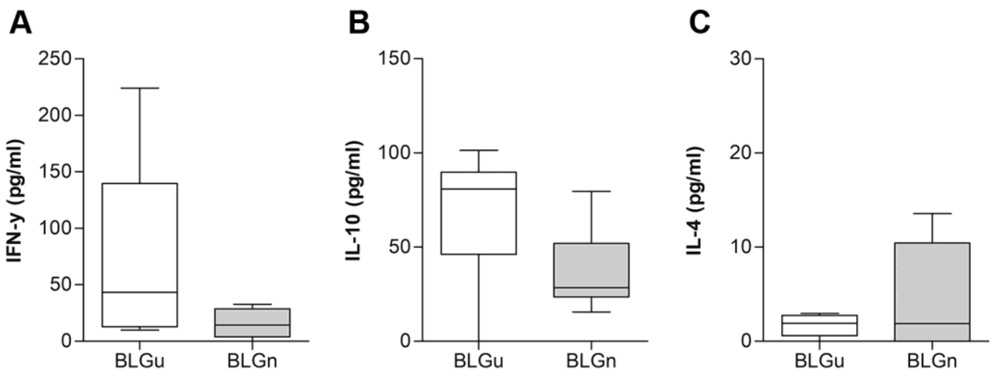

Supporting Information 5

Effect of re-stimulation of spleen cells with BLGn and influence of prior feeding on cytokine production in allergic mice. Isolated spleen cells from allergic mice fed with BLGu or BLGn were stimulated with BLGn to evaluate cytokine production upon allergen encounter. Baseline levels were subtracted. We could not detect significant differences between feeding groups for **(A)** IFN-γ (unpaired t test with Welch correction) or **(B)** IL-10 (unpaired t test). **(C)** IL-4 remained mostly below detection limit of the assay used and did not show any differences (t test with Welch's correction). BLG, beta-lactoglobulin; BLGn, nitrated BLG; BLGu, untreated BLG
